# An auditory health program for neonates in ICU and/or intermediate care settings

**DOI:** 10.5935/1808-8694.20130130

**Published:** 2015-10-08

**Authors:** Maria Francisca Colella-Santos, Edi Lucia Sartorato, Tatiana Guilhermino Tazinazzio, Maria de Fátima de Campos Françozo, Christiane Marques do Couto, Arthur Menino Castilho, Izilda Rodrigues Machado Rosa, Maria Cecilia Marconi Pinheiro Lima, Sérgio Tadeu Martins Marba

**Affiliations:** a Post-doctoral degree holder (Professor, Coordinator of the Speech and Hearing Therapy Program, UNICAMP); b Associate Professor (Researcher, Adjunct Director, UNICAMP Molecular Biology and Genetics Center); c MSc. (Speech and Hearing Therapist, Neonatology Service, Prof. Dr. José Aristodemo Pinotti Women's Hospital - CAISM/UNICAMP); d PhD (Professor, Speech and Hearing Therapy Program, Medical Sciences School, UNICAMP); e PhD, M.D., ENT, Department of Ophthalmology and Otorhinolaryngology, Unicamp (M.D., ENT, Department of Ophthalmology and Otorhinolaryngology, Medical Sciences School, UNICAMP); f PhD (Director, neonatology Division, CAISM/UNICAMP); g Associate Professor (Director, Research Support and Assistance Division, Prof. Dr. José Aristodemo Pinotti Women's Hospital - CAISM/UNICAMP). State University of Campinas

**Keywords:** hearing loss, hearing tests, infant, screening

## Abstract

Auditory screening and early identification and management of patients with hearing loss improve the development prospects of infants.

**Objective:**

To analyze the outcomes produced by an Auditory Health Program in neonates managed in an intensive care unit.

**Method:**

This prospective cross-sectional study enrolled neonates referred to the neonatal care unit at hospital CAISM/Unicamp with stays lasting for 48 hours and more within a period of 13 months.

Automated monitoring of brainstem auditory evoked potentials was used in the auditory screening of neonates at the time of discharge. Children with poor BAEPs were sent to undergo audiological, otorhinolaryngological, and genetic tests.

**Results:**

Auditory screening was performed for 84.7% of the live births; 39.7% were screened at 30 days or more of age. Diagnostic tests revealed that 63.8% of the children had normal hearing. Incidence of hearing loss was 4%; sensorineural hearing loss was observed in 1.4% of the subjects; 0.24% had auditory neuropathy spectrum disorder; and 2.2% had conductive hearing loss.

**Conclusion:**

Neonatal auditory screening was not offered universally, and nor was it carried out, in many cases, within the child's first month of life. Screening must be performed before neonates are discharged and in more than one stage. A high incidence of hearing loss was observed.

## INTRODUCTION

Mortality rates in high-risk neonates have progressively dropped due to the advances in technology in neonatal intensive care units (ICU), medical devices, drugs, and the growing number of medical professionals specialized in this area.

The advances in neonatology have led to higher survival rates among preterm low-weight babies. However, health care workers have been increasingly concerned with the quality of life these neonates will enjoy, as sequelae often set in after discharge.

Hearing loss is a frequent alteration in children seen in neonatal ICUs, with incidences ranging between 1% and 4% in this population1., 2..

Sensory deprivation consequent to hearing loss, specifically in the early stages of language acquisition, introduces significant barriers to the overall development of the affected children^3^. Hearing loss may lead to deficits in language acquisition and cognitive, intellectual, cultural, and social impairment.

Universal neonatal auditory screening has been recommended as the main strategy to reduce the age at which hearing loss is diagnosed^1^. Early diagnosis followed by medical intervention and speech and hearing therapy enable children to have contact with sound stimuli while there still is significant plasticity in their central nervous systems (in their first year of life), thus allowing the development of nerve connections and improved outcomes in auditory rehabilitation and the overall development of children with hearing loss4., 5..

The Neonatal Auditory Health Program encompasses a set of actions involving universal auditory screening for neonates accompanied by speech and hearing, otorhinolaryngological, and genetic tests for the subjects singled out during screening or speech and hearing tests. When performed early on, these procedures may improve patient development.

Few studies have addressed the outcomes of children in need of intensive care.

Therefore, this study aimed to analyze the outcomes produced by an Auditory Health Program including auditory screening and hearing loss diagnosis for neonates treated in an intensive care unit and/or intermediate care setting at Prof Dr. José Aristodemo Pinotti CAISM Hospital.

## METHOD

This cross-sectional prospective trial was approved by the Ethics in Research Committee School of Medical Sciences, University of Campinas (FCM/Unicamp) and was given permit 1.085/2009. Subjects were enrolled voluntarily and their parents signed an informed consent term for their participation.

The study included neonates seen in the ICU and/or intermediate care center at CAISM Hospital with stays lasting for 48 hours and more within a period of 13 months, between March of 2011 and March of 2012. Children born in other services, and patients who died or failed to complete all the stages of the study were excluded. Initially, the risk indicators on the JCIH and COMUSA1., 2. were analyzed for each patient, along with their personal identification information, data on their birth status, and patient charts. Automated monitoring of brainstem auditory evoked potentials (Accuscreen - GN Otometrics) was used in the auditory screening of neonates as they were about to be discharged. Auditory screening sessions were held four times a week - on Mondays, Wednesdays, Thursdays, and Fridays - in the newborn care room. Referred infants and individuals unable to be tested were screened after discharge in appointments set by the medical team. Patients responding to clicks of 35 dB bilaterally were considered to have performed well. The children who failed to respond were referred for audiological, otorhinolaryngological, and genetic testing.

Audiological assessment was carried out at the pediatric audiological diagnosis laboratory at Unicamp Cepre Pediatric Diagnostics Lab, FCM, Unicamp in a sound proof room. The test included the following steps: interview, middle ear examination, and BAEPs (i.e., electrophysiological thresholds and auditory pathway integrity), in addition to transient otoacoustic emissions (TOAEs) and/or distortion product otoacoustic emissions (DPOAEs). The patients were sleeping during the administration of the test procedures.

Electrophysiological thresholds, as well as auditory pathway integrity, were assessed through BAEPs on an Interacoustics Eclipse EP 25 with patients wearing ear buds. Auditory pathway integrity was tested with non-variable 80-dB clicks, to allow the assessment of the auditory pathway to the brainstem and identify possible alterations in its course. Electrophysiological thresholds were captured by offering patients stimuli at decreasing intensities until the V wave was triggered, using click and tone burst stimuli at 500 and 1,000 Hz. Stimulation was repeated twice to verify the reproducibility of the tracings and the presence of response. Responses were captured through surface electrodes with electrolytic paste placed on the right and left mastoid and on the frontoparietal position. Patients had their skin cleaned with abrasive paste on the sites on which electrodes were placed. The following parameters were analyzed: presence of waves I, III, and V; absolute latency of waves I, III, and V; interpeak latency I-V, I-III, III-V; amplitude of wave V in relation to amplitude of wave I; interaural differences in I-V interpeak latency or wave V latency. TOAEs and DPOAEs were captured using device ILO 292 USBII.

Middle ear status was assessed through otoscopic examination (to verify whether patients were able to undergo impedance testing), tympanogram tracing, and ipsilateral acoustic reflex from 500 to 4,000 Hz, with the tone in the transducer set at 1,000 Hz. Impedance tests were carried out on a 235H-Interacoustics device.

The responses obtained in the tests were recorded in a sheet. Normal hearing was attributed to infants with electrophysiological thresholds equal to or under 30 dB and absolute and interpeak latencies within the values expected for the subjects' ages and responses within expected ranges for the remaining test procedures.

Patients with altered audiological test results were referred to an ENT physician for physical examination and imaging tests.

The team's geneticist tested neonates underperforming in the screening tests by collecting DNA from the oral mucosa according to the protocol adopted in Laboratory Unicamp Cepre Pediatric Diagnostics Lab, FCM, Unicamp. DNA samples were collected by the examiner after the completion of the auditory tests. Oral mucosa DNA was used to analyze mutation 35delG by AS-PCR as standardized by the Human Molecular Genetics Lab at CBMEG (patent n^o^ P10005340-6; testing method for hearing loss of genetic origin). PCR was used to analyze deletions D(GJB6-D13S1830) and Δ(GJB6-D13S1854) using previously described primers^6^. Mitochondrial mutations were analyzed through the amplification of DNAmt fragments from gene 12S rRNA to detect mutations A1555G, C1494T, A827G, using previously described primer pairs. The product of amplification was submitted to restriction analysis to detect mutations.

The data collected from patient charts and the results from auditory screening tests were recorded in an information system. These data sets were used to build descriptive charts and to further statistically analyze the results.

## RESULTS

A total of 526 children stayed in the ICU and/or intermediate care unit at CAISM/Unicamp University Hospital for at least 48 hours between March of 2011 and March of 2012. Thirty-seven individuals died, yielding 489 live births; 53.2% were boys, 67% were preterm births, 44% weighed less than 2,000 g, and 20% had very light weight at birth (< 1500g); 59% had two to four risk factors for hearing loss.

Table 1 shows the results of neonatal auditory screening tests.

**Table 1:** Auditory screening results of the neonates in the CAISM neonatal ICU and intermediate care facility.

Auditory Screening	n	%
Yes	414	84.7
No	75	15.3
Total	489	100
Yes/Pass	337	81.4
Failed 35 dB	4	0.96
Failed 40 dB	19	4.6
Failed 45 dB	54	13.04

No statistically significant difference was seen when pass/fail statuses and gender (*p* = 0.3963), gestational age (*p* = 0.3448), weight (*p* = 0.3291), number of risk factors (*p* = 0.4826), or age after birth at which the tests were carried out (*p* = 0.1654) were analyzed. Among risk factors, only hyperbilirubinemia was statistically significant in relation to the outcome of the auditory screening tests. Two thirds (66.7%) of the children with this risk factor failed auditory screening *(p* = 0.0002).

Table 2 shows the number of days after birth at which the individuals underwent auditory screening.

**Table 2:** Age (days) at which screening tests were carried out.

Age	n	%
< 30 days	243	60
30 to 60 days	85	21
> 60 days	77	19

Mean = 36.6 days.

The subjects who failed screening tests were referred for diagnosis. Fifty-seven percent of the individuals (44/77) completed the required diagnostic tests. The other patients were excluded from the program for not having completed the process after four attempts of scheduling visits for them.

Table 3 shows the results from the combined analysis of audiological and otorhinolaryngological tests.

**Table 3:** Audiological and otorhinolaryngological diagnosis of the children who failed the auditory screening tests.

Diagnosis	n	%
Normal hearing	28	63.8
Conductive hearing loss	9	20.4
Sensorineural hearing loss	6	13.6
Auditory neuropathy spectrum disorder	1	2.2
Total	44	100

Mutation 35delG in the connexin 26 gene (GJB2) or deletions Δ(GJB6-D13S1830) and Δ(GJB6-D13S1854) in gene GJB6 were not seen in the genetic tests performed on the 44 patients who failed auditory screening tests. Two children had mutation A1555G in mitochondrial gene MTRNR. These children had normal hearing. Table 4 shows the incidence of hearing loss in the studied sample.

**Table 4:** Main study findings for the enrolled population.

Results	n	%
Passed Screening	337	81.4
Failed Auditory	77	18.6
Total	414	100
Auditory diagnosis Normal	28	63.8
CHL	9	20.4
SNHL	6	13.6
ANSD	1	2.2
Total	44	100
Incidence CHL	9/414	2.2
SNHL	6/414	1.4
ANSD	1/414	0.24
CHL + SNHL + ANSD	16/414	3.84

CHL: Conductive hearing loss; SNHL: Sensorineural hearing loss; ANSD: Auditory neuropathy spectrum disorder.

No statistically significant difference was seen when diagnosis of normal hearing and hearing loss were considered vis-à -vis gender (*p* = 0.3477), gestational age (*p* = 0.2523), weight (*p* = 0.2491), and number of risk factors (*p* = 0.067). Statistically significant difference was seen (*p* = 0.002) between failed auditory screening tests for 35, 40, and 45 dB and patient diagnosis. Most of the children (90.9%) who failed the test at 40 dB had normal hearing.

## DISCUSSION

Neonatal auditory screening is the most significant tool in the early detection of hearing loss. The procedure must be quick, simple, and able to select the patients with the highest probability of having alterations for the tested function^7^. Neonates in ICU settings usually have more than one risk factor, many of which conducive to hearing loss. Consequently, the incidence of hearing loss in this population is higher - from 1% to 4%^8^. Automated BAEP monitoring is a recommended screening procedure that assesses the auditory system to the brainstem and uses statistical criteria to pass or fail patients in the test. Patients pass or fail based on their response to click stimuli. The test does not allow wave visualization^9^. It is a highly sensitive (test ability to identify hearing loss) and specific (test ability to identify individuals as having normal hearing) method^10^. Some authors have rated it as reasonably specific, as tests misses out on rare ascending hearing losses and conductive hearing loss^11^.

Auditory screening was done in 84.7% of the 489 newborns who stayed in the neonatal ICU for 48 hours and more (Table 1). The goal was to offer universal auditory screening, as described in the JCIH and COMUSA1., 2.. However, some factors made it impossible to reach a rate of 95% or greater of screened infants. The CAISM is a tertiary care university hospital located in the metropolitan areas of Campinas and Piracicaba, an area encompassing over 60 municipalities, and a reference center for female and neonate health care prepared to handle high risk pregnancies due to maternal or fetal disease. In order to meet the significant demand for ICU services, neonates are often referred to other centers for care. This particularity pertaining to our service made it impossible for some neonates to undergo auditory screening, as they were using devices that interfered with the equipment needed to perform screening tests.

In many cases screening was not indicated for the risk neonates were of having their auditory systems impaired due to ototoxic drugs or factors connected with the transference of patients to other institutions. The study's staff attempted to schedule the patients initially unavailable for screening to undergo testing at a later time, but many refused to show up despite our efforts. Many neonates discharged during weekends - when screening tests were not offered - were also lost.

In order to increase the number of screened neonates to cover 95% or more of them, it is recommended, whenever possible, to perform screening tests before patients are discharged to minimize the possibility of subjects missing their scheduled visits. To do so, it is recommended that a speech and hearing therapist be hired for at least 20 hours a week and to work on call during weekends. Another factor that may improve compliance with auditory screening is providing counseling to the families of the neonates, so they are informed of the importance of hearing in the development of speech and language and the role of auditory screening in the early detection of hearing loss, and the relevance of showing up in the scheduled date to have their children undergo the screening tests. A poll done with 35 parents and guardians of pediatric ICU patients revealed that approximately 80% of the them had never heard about auditory screening, its use, or the importance of hearing^12^. Other studies will be carried out to verify whether auditory health education for parents and expecting mothers could increase program compliance.

In Brazil, several studies have failed to attain 95% compliance in neonatal auditory screening13., 14., 15.. No epidemiological studies were found on overall compliance rates of auditory screening programs in Brazil, but studies are known to be concentrated in large centers and maternity hospitals, albeit to a much lesser degree. Federal Law 12303 as of August 2, 2010^16^, which made it mandatory for all hospitals and maternity hospitals to offer evoked otoacoustic emissions testing free of charge to all children born in their premises, and the Guidelines for Neonatal Auditory Care and Screening published in 2012^17^ are expected to positively change this scenario.

Most of the children passed the auditory screening test, i.e., they responded to 35-dB bilateral click stimuli on BAEP testing. Overall failure rate was 18.6%, which was above the rates published in the literature; most subjects failed at 45 dB (Table 1). Such high failure rate was due to the lack of experience the team had using a new testing device, and possibly other factors as well, such as the high rate at which the device presented click stimuli (55 Hz) and the automated response analysis feature, as in preterm babies latencies may be different than the norm. Additionally, the difficulties placing the electrodes and fitting the ear buds in the narrow ear canals of the children may have increased the number of failed test results.

Future studies will be carried out to eliminate the impact of lack of experience with the device so that other possible causes of failure are singled out.

An analysis of the age at which auditory screening was carried out revealed that 21.1% of the children were tested between 30 and 60 days of age and 18.6% after 60 days of age (Table 2). Auditory screening should be carried out close to the time of discharge, after newborns have overcome the possible adverse events connected to prenatal, perinatal, and post-natal care and when they no longer need to be in an incubator, on life support, or medications. According to the JCIH^1^ and the COMUSA^2^, screening should be performed by the first month of life, hearing loss should be diagnosed by the third month of life, and fitting of hearing aids should occur by the sixth month of life. These recommendations do not apply to many of the neonates staying at ICUs, as they often require hospitalization for over 60 days.

The children who passed the screening tests and had risk factors for progressive and/or late onset hearing loss were referred for hearing and language monitoring at 6, 12, 18, and 24 months of age. This measure aimed to detect possible cases of ascending or conductive hearing loss in patients who passed the screening tests.

The patients who failed the screening tests were referred for diagnosis. Audiological and otorhinolaryngological tests revealed that 63.8% (28/44) of the subjects had normal hearing. Hearing loss was observed in 36% (16/44) of the children; 20.4% (9/44) had conductive hearing loss; 13.6% (6/44) had sensorineural hearing loss; and 2.2%(1/44) had auditory neuropathy spectrum disorder (Table 3).

Most of the children who failed the screening tests had normal results in the hearing tests applied. There was a high rate of false positive results, i.e., children failing the auditory screening tests were deemed to have normal hearing in diagnostic evaluation. Automated monitoring of BAEPs in auditory screening has been claimed to reduce the need to refer patients to diagnostic assessment. The reduced rates reported in the literature are based on a two-stage auditory screening process (test-retest)18., 19.. More familiarity with the screening equipment, combined to retesting, may substantially reduce the rate of false positive results and the number of patients lost in follow-up and normal results, in addition to minimizing parental stress and anxiety with the outcomes of auditory screening tests. National and international science committees do not support the retesting of populations at risk; rather, they should be immediately referred to diagnostic examination1., 2..

Forty-three percent of the patients referred to diagnostic evaluation were lost in follow-up. Patient evasion in auditory health programs is a global reality. Compliance to auditory health programs must be improved to allow the early detection of individuals with hearing loss. Factors such as low number of prenatal care visits (one to three), having more than one child, being a single parent, and mothers with low levels of education impact infant health program compliance rates. It has been recommended that topics concerning the relevance of hearing for the development of children and the possibility of detecting early cases be discussed during prenatal care visits, so that parents become aware of the facts to consider on infant deafness and its adverse impacts. The importance of neonatal auditory screening should be more communicated to patients, health care workers, and pediatricians in particular, as neonates are part of the audience they serve, so as to encourage patient attendance to screening tests.

The incidence of hearing loss in the studied sample was of approximately 4%; 1.4% of the subjects had sensorineural hearing loss, 0.24% had auditory neuropathy spectrum disorder, and 2.2% had conductive hearing loss (Table 4). The incidence rates published in the literature usually cover sensorineural hearing loss. Reported incidence rates among neonates in ICU settings range between 1% and 4%1., 2., 11., 20., 21., as also found in our study. The same was observed for auditory neuropathy spectrum disorder, with rates ranging from 0.2% to 4%22., 23., 24..

Genetic assays showed patients were normal for mutation 35delG in the connexin 26 (GJB2) gene. Although none of the individuals had mutation 35delG, it is present in 70% of the individuals with hearing loss in whom gene GJB2 is involved. A prevalence of 0.97% has been reported in Brazil for mutation 35delG, a rate of 1:103 heterozygotes, in a screening initiative carried out with 620 neonates in the region of Campinas^25^ In addition to gene GJB2, deletions Δ(GJB6-D13S1830) and Δ(GJB6-D13S1854) on gene GJB6 were screened for. None of the individuals enrolled in this study had the aforementioned deletions. Screening was also done for mitochondrial mutation A1555G, with described associations with hearing loss and use of aminoglycosides. Two children had mutation A1555G on mitochondrial gene MTRNR, and both had normal hearing. The negative test results for the studied gene mutations reduce the empirical risk related to a genetic etiology for hearing loss.

The children diagnosed with conductive hearing loss are being followed by the team's ENT physician and, whenever needed, they are referred for audiological evaluation. They will be followed by a multidisciplinary team, as the impact of conductive hearing loss upon the development of hearing, language, and school performance has been established. The patients with sensorineural hearing loss are currently having hearing aids fitted (conventional aids or cochlear implants) and speech and hearing rehabilitation.

The correlation between the studied variables and failure in auditory screening tests was not statistically significant. When risk factors are considered, only hyperbilirubinemia significantly affected the outcome of auditory screening tests. Diagnosis was statistically correlated with failing screening tests at 35, 40, 45 dB. Most of the children (90.9%) who failed the tests at 40 dB had normal hearing. More studies will be carried out to increase the size of the sample and verify this finding. The low number of children diagnosed with hearing loss may have affected the test results. The literature indicates higher incidences of hearing loss in children weighing 1,500 g and less and preterm babies26., 27., 28..

## CONCLUSION

The analysis of the results of this study indicated it was not possible to offer universal neonatal auditory screening in the studied sample or to screen patients within their first 30 days of life, given the long time for which neonates stay in intensive care. Whenever possible, auditory screening should be carried out before discharge. One-stage auditory screening led to a significant number of false-positive results. The overall incidence of hearing loss was 4%, including cases of sensorineural and conductive hearing loss, and auditory neuropathy spectrum disorder.

## References

[1] American Academy of Pediatrics, Joint Committee on Infant Hearing (2007). Year 2007 position statement: Principles and guidelines for early hearing detection and intervention programs. Pediatrics.

[2] Lewis DR, Marone SA, Mendes BC, Cruz OL (2010). Nóbrega Md. Multiprofessional committee on auditory health: COMUSA. Braz J Otorhinolaryngol.

[3] Sininger YS, Doyle KJ, Moore JK. (1999). The case for early identification of hearing loss in children. Auditory system development, experimental auditory deprivation, and development of speech perception and hearing. Pediatr Clin North Am.

[4] Durieux-Smith A, Fitzpatrick E, Whittingham J. (2008). Universal newborn hearing screening: a question of evidence. Int J Audiol.

[5] Hyde ML. (2005). Newborn hearing screening programs: overview. J Otolaryngol.

[6] del Castillo FJ, Rodríguez-Ballesteros M, Alvarez A, Hutchin T, Leonardi E, de Oliveira CA (2005). A novel deletion involving the connexin-30 gene, del(GJB6-d13s1854), found in trans with mutations in the GJB2 gene (connexin-26) in subjects with DFNB1 non-syndromic hearing impairment. J Med Genet.

[7] Northern JL, Downs MP. (2005). Audiçã o em crianças.

[8] Erenberg A, Lemons J, Sia C, Trunkel D, Ziring P. (1999). Newborn and infant hearing loss: detection and intervention. American Academy of Pediatrics. Task Force on Newborn and Infant Hearing, 1998–1999. Pediatrics.

[9] Benito-Orejas JI, Ramírez B, Morais D, Almaraz A, Fernández-Calvo JL. (2008). Comparison of two-step transient evoked otoacoustic emissions (TEOAE) and automated auditory brainstem response (AABR) for universal newborn hearing screening programs. Int J Pediatr Otorhinolaryngol.

[10] Hall JW, Smith SD, Popelka GR. (2004). Newborn hearing screening with combined otoacoustic emissions and auditory brainstem responses. J Am Acad Audiol.

[11] Angrisani RMG, Suzuki MR, Pifaia GR, Testa JR, Sousa EC, Gil D (2012). PEATE automático em recém-nascidos de risco: estudo da sensibilidade e especificidade. Rev CEFAC.

[12] Eusebio D, Colella-Santos MF. (2012). Triagem auditiva neonatal: orientaçã o aos pais. [Monografia de conclusão de curso de graduaçã o].

[13] Scaziotta MACM, Andrade IFC, Lewis DR. (2012). Programa de triagem auditiva seletiva em crianças de risco em um serviço de saúde auditiva de São Paulo. Rev CEFAC.

[14] Boscatto SD, Machado MS. (2013 May 05). Teste da orelhinha no hospital São Vicente de São Paulo: levantamento de dados. Rev CEFAC. [serial on the Internet].

[15] Onoda RM, Azevedo MF, Santos AM. (2011). Neonatal Hearing Screening: failures, hearing loss and risk indicators. Braz J Otorhinolaryngol.

[16] Brasil. Presidência da República. Lei 12.303/2010 (Lei Ordinária) 02/08/2010 [Acessado em 15 de outubro de 2013]. Disponível em: http://www.planalto.gov.br/ccivil_03/_Ato2007-2010/2010/Lei/L12303.htm

[17] Diretrizes de Ateçã o da Triagem Auditiva neonatal [Acessado em 15 de outubro de 2013]. Disponível em: http://bvsms.saude.gov.br/bvs/publicacoes/diretrizes_atencao_triagem_auditiva_neonatal.pdf

[18] Freitas VS, Alvarenga KF, Bevilacqua MC, Martinez MAN, Costa OA. (2009). Análise crítica de três protocolos de triagem auditiva neonatal. Pró-Fono.

[19] Lin HC, Shu MT, Lee KS, Lin HY, Lin G. (2007). Reducing false positives in newborn hearing screening program: how and why. Otol Neurotol.

[20] Uus K, Bamford J. (2006). Effectiveness of population-based newborn hearing screening in England: ages of interventions and profile of cases. Pediatrics.

[21] Connolly JL, Carron JD, Roark SD. (2005). Universal newborn hearing screening: are we achieving the Joint Committee on Infant Hearing JCIH) objectives? Laryngoscope.

[22] Ngo RY, Tan HK, Balakrishnan A, Lim SB, Lazaroo DT. (2006). Auditory neuropathy/auditory dys-synchrony detected by universal newborn hearing screening. Int J Pediatr Otorhinolaryngol.

[23] Stein LK, Tremblay K, Pasternak J, Banerjee S, Lindemann K, Kraus N. (1996). Brainstem abnormalities in neonates with normal otoacoustic emissions. Semin Hear.

[24] Berlin C, Hood L, Rose K. (2001). On renaming auditory neuropathy as auditory dys-synchrony. Audiol Today.

[25] Sartorato EL, Gottardi E, de Oliveira CA, Magna LA, Annichino-Bizzacchi JM, Seixas CA (2000). Determination of the frequency of the 35delG allele in Brazilian neonates. Clin Genet.

[26] Cristobal R, Oghalai JS. (2008). Hearing loss in children with very low birth weight: current review of epidemiology and pathophysiology. Arch Dis Child Fetal Neonatal Ed.

[27] Robertson CM, Howarth TM, Bork DL, Dinu IA. (2009). Permanent bilateral sensory and neural hearing loss of children after neonatal intensive care because of extreme prematurity: a thirty-year study. Pediatrics.

[28] Synnes AR, Anson S, Baum J, Usher L. (2012). Incidence and pattern of hearing impairment in children with ≤ 800 g birthweight in British Columbia, Canada. Acta Paediatr.

